# Combinatorial Approaches to Viral Attenuation

**DOI:** 10.1128/mSystems.00046-18

**Published:** 2018-07-31

**Authors:** Matthew L. Paff, Benjamin R. Jack, Bartram L. Smith, James J. Bull, Claus O. Wilke

**Affiliations:** a Department of Integrative Biology, The University of Texas at Austin, Austin, Texas, USA; b Institute for Cellular and Molecular Biology, The University of Texas at Austin, Austin, Texas, USA; Dartmouth College

**Keywords:** bacteriophages, codon deoptimization, promoter knockout, viral attenuation

## Abstract

Attenuated viruses have numerous applications, in particular in the context of live viral vaccines. However, purposefully designing attenuated viruses remains challenging, in particular if the attenuation is meant to be resistant to rapid evolutionary recovery. Here we develop and analyze a new attenuation method, promoter ablation, using an established viral model, bacteriophage T7. Ablation of promoters of the two most highly expressed T7 proteins (scaffold and capsid) led to major reductions in transcript abundance of the affected genes, with the effect of the double knockout approximately additive of the effects of single knockouts. Fitness reduction was moderate and also approximately additive; fitness recovery on extended adaptation was partial and did not restore the promoters. The fitness effect of promoter knockouts combined with a previously tested codon deoptimization of the capsid gene was less than additive, as anticipated from their competing mechanisms of action. In one design, the engineering created an unintended consequence that led to further attenuation, the effect of which was studied and understood in hindsight. Overall, the mechanisms and effects of genome engineering on attenuation behaved in a predictable manner. Therefore, this work suggests that the rational design of viral attenuation methods is becoming feasible.

**IMPORTANCE** Live viral vaccines rely on attenuated viruses that can successfully infect their host but have reduced fitness or virulence. Such attenuated viruses were originally developed through trial and error, typically by adaptation of the wild-type virus to novel conditions. That method was haphazard, with no way of controlling the degree of attenuation or the number of attenuating mutations or preventing evolutionary reversion. Synthetic biology now enables rational design and engineering of viral attenuation, but rational design must be informed by biological principles to achieve stable, quantitative attenuation. This work shows that in a model system for viral attenuation, bacteriophage T7, attenuation can be obtained from rational design principles, and multiple different attenuation approaches can be combined for enhanced overall effect.

## Abstract (precis)

Live viral vaccines rely on attenuated viruses that can successfully infect their host but have reduced fitness or virulence. Such attenuated viruses were originally developed through trial and error, typically by adaptation of the wild-type virus to novel conditions. That method was haphazard, with no way of controlling the degree of attenuation or the number of attenuating mutations or preventing evolutionary reversion. Synthetic biology now enables rational design and engineering of viral attenuation, but rational design must be informed by biological principles to achieve stable, quantitative attenuation. This work shows that in a model system for viral attenuation, bacteriophage T7, attenuation can be obtained from rational design principles, and multiple different attenuation approaches can be combined for enhanced overall effect.

## INTRODUCTION

Live viral vaccines are in wide use and have been immensely effective. A classic example, the Sabin oral polio vaccine (OPV), is largely responsible for eradicating polio in the majority of the world (1, 2). Most live vaccines have been developed as “attenuated” or genetically weakened versions of their wild-type counterparts. Use of attenuated vaccines has a long history, and out of necessity in an era before genetic engineering, methods of achieving attenuation were empirical, adapting the wild-type virus to novel conditions in the hope that growth was retarded in the original host (3–5). Despite many successes, this method was haphazard, often failing to attenuate or producing unstable attenuations that quickly evolved back to high virulence. The most dramatic example of vaccine reversion, that of OPV, resulted in many vaccine-derived cases of poliomyelitis and circulation of vaccine-derived polioviruses (5–7).

Advances in synthetic biology and genome engineering are being utilized in conjunction with computational and modeling approaches (8, 9) to now enable rational design and facile creation of attenuated viruses, with the hope of avoiding problems encountered by classic methods. Strategies for engineered viral attenuation include codon modification either through deoptimization (10–15) or optimization (16), targeting of viral mRNA folding structure (17), altered fidelity of RNA-dependent RNA polymerase (RNAP) replication in RNA viruses (18–21), self-attenuating microRNAs (miRNAs) (22), genome rearrangements, and gene deletions (reviewed in references 4, 23, and 24). Despite the ease of engineering attenuation and the improved success of new methods in suppressing viral growth in the short term, the underlying molecular bases of attenuation often remain cloudy. Against this drawback, however, the rapidly increasing knowledge base of molecular virology points to a future of highly predictable attenuation methods, with the ability to control levels of attenuation quantitatively while also blocking evolutionary reversion of vaccine strains.

Here we build upon an already expansive body of work on attenuation methods in a bacterial virus, T7, to develop and analyze a new method and evaluate that method alone and in combination with a previous attenuation (12, 25). T7 is one of the most thoroughly studied viruses, and several engineering-based attenuation methods have been tested for initial effects and robustness to evolutionary reversion: gene deletion, genome rearrangement, and codon deoptimization (23). The new method considered here, promoter knockout, should reduce fitness quantitatively according to the numbers of promoters knocked out and also depending on the identities of the promoters.

Fitness reduction by promoter knockout is expected to have a clear predictable molecular basis in T7: major reduction of transcript abundance of an essential gene. The effect of this reduction is to cause an imbalance in gene expression that should in turn cause a mismatch between the relative abundances of proteins and the needs dictated by stoichiometric demands of virus assembly. Promoter knockouts in T7 do not abolish gene activity and thus should not be lethal, as transcripts initiated from upstream promoters extend across many genes—there is only one terminator for T7 RNA polymerase in the genome, and even it terminates incompletely. Thus, we expect both that promoter knockouts will reduce fitness and that promoter knockouts can be combined to allow progressively greater fitness reduction (attenuation), a prediction we test by comparing single and double knockouts. We also combine promoter knockouts with a previously implemented attenuation method, codon deoptimization, to evaluate possible synergy of mechanisms.

Depending on the design, the attenuation of a virus may be subject to evolutionary recovery—higher fitness—as the attenuated virus grows and is selected for faster growth. Predicting and blocking evolutionary recovery are secondary goals in attenuation design. With promoter knockouts, recoveries are predicted to be thwarted for two reasons. First, redressing the imbalance in gene expression caused by a knockout should require re-evolving a promoter; merely upregulating entire suites of genes (or increasing genome synthesis to increase templates for transcription) would not restore balance. Second, stepwise re-evolving the suite of the ~23 bases comprising a T7 promoter should be a slow process, too slow to observe in an experimental adaptation.

In the course of creating our attenuated T7 strains, we inadvertently introduced, discovered, and explored an unintended consequence of the engineering, leading to a third but unstable mechanism of attenuation. In all, the multitiered approach to attenuation offered here suggests that our understanding of mechanisms is advancing to the point that attenuation and its evolutionary stability are becoming broadly predictable.

## RESULTS

### The model system: T7.

Bacteriophage T7 contains approximately 60 genes, 19 of which are known to be essential (26, 27). Gene expression occurs linearly and in a temporal fashion, with 3 distinct classes of genes, I, II, and III, defined as early, middle, and late, respectively. Class I genes enter the cell first and are expressed by the Escherichia coli host RNA polymerase (RNAP). T7 carries its own RNAP gene (gene *1*) which is the last of the class I genes (26, 27). Class II (DNA metabolism) and III (morphogenesis) genes are expressed using T7 RNAP from 17 different phage promoters, and class II promoters have slightly different sequences than class III promoters. Initially, gene expression occurs primarily from class II promoters. Production of gp3.5 (lysozyme) results in a T7 RNAP-lysozyme complex that shifts preference for binding class III promoters, the first of which lies upstream of gene *6.5*. Expression of class II genes still occurs, but at a reduced rate. The majority of transcripts are polycistronic, as there is only one known T7 RNAP terminator (*T*ϕ), located immediately downstream of gene *10A*.

The most highly expressed genes in T7 are those coding for the scaffold and the major capsid proteins (genes *9* and *10A*, respectively) (25, 26). Both genes have their own class III promoters (ϕ9 and ϕ10), located immediately upstream, which drive the majority of gene *9* and *10A* expression during the T7 life cycle. Since the only T7 terminator (*Tϕ*) is located after gene *10A*, we are able to knock out promoters upstream while maintaining phage viability. We ablated promoters ϕ9 and ϕ10 and replaced them with arbitrary sequences so that the promoter function was abolished but the same length of DNA was maintained.

We evaluated the effects of promoter knockouts in three different genetic backgrounds (Table 1), one of which was accidental: (i) the wild type (wt), (ii) a strain in which gene *10* was engineered with noncoding changes in nearly 200 codons to deoptimize gene expression (10_deop_), and (iii) a strain in which the stop codon for gene *8* was abolished (8_Δstop_), such that the gene *8* transcript encodes an additional 25-amino-acid readthrough product. The last of these backgrounds stemmed from an unintended consequence of our first attempt to ablate the ϕ9 promoter, not appreciating that the promoter contained the stop codon for gene *8*. Given that we understand this unintended consequence, even if *post hoc*, we can use the engineering to represent a third background (Table 1). The testing of promoter knockouts in backgrounds with and without alternative attenuating mechanisms allows us to study the effects of combining different mechanisms of attenuation.

**TABLE 1:** T7 knockout strains

Background genotype	Description	Knockout strains
wt	Wild type	Δϕ9_wt_, Δϕ10_wt_, Δϕ9/ϕ10_wt_^a^
10_deop_	Codon-modified gene *10* with 10% preferred codon usage	Δϕ9_10_deop__, Δϕ10_10_deop__, Δϕ9/ϕ_10_deop__^a^
8_Δstop_	Stop codon for gene *8* abolished; generates 25-amino-acid readthrough product	Δϕ9_8_Δstop__,^a^ Δϕ9/ϕ10_8_Δstop__^a^

a Strains used for long-term evolution experiments. The resulting evolved strains are notated with the “evo-” prefix (e.g., evo-Δϕ9/ϕ10_wt_) throughout the article.

### Diminishing returns in fitness reduction observed for combined attenuations.

**FIG 1:**
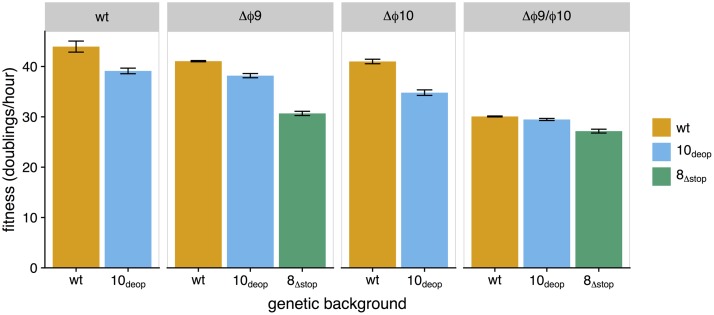
Initial fitness of promoter knockout strains. Fitness (measured as doublings per hour) was quantified for single (Δϕ9 and Δϕ10) and double (Δ*ϕ*9/ϕ10) promoter knockout strains engineered into three different genetic backgrounds (wild type [wt], orange; gene *10* codon deoptimized, blue; and an abolished gene *8* stop codon, green). Comparison to wild type indicates significantly reduced fitness (10_deop_, *P* = 0.0297; Δϕ9_10_deop__, *P* = 0.0250; Δϕ9_8_Δstop__, *P* = 0.00436; Δϕ10_10_deop__, *P* = 0.00755; Δϕ9ϕ10_wt_, *P* = 0.00718; Δϕ9/ϕ10_10_deop__, *P* = 0.00586; Δϕ9/ϕ10_8_Δstop__, *P* = 0.00296; paired *t* tests) in all but 2 strains (Δϕ9_wt_, *P* = 0.0889; Δϕ10_wt_, *P* = 0.0749; paired *t* tests). Additionally, no significant difference was detected between Δϕ9_10_deop__ and wt_10_deop__ (*P* = 0.253, paired *t* test).

To quantify the extent of attenuation for our promoter knockout strains, we measured fitness in each strain (Fig. 1). The measure of fitness used here is the growth rate of the phage population when hosts are not limiting, presented as doublings per hour. All knockout strains exhibited reduced fitness relative to their unaltered backgrounds, although the effect was not statistically significant in two cases (Fig. 1; see Table S1 in the supplemental material). Within the group of strains containing individual promoter knockouts, Δϕ9_8_Δstop__ had the largest fitness reduction in terms of magnitude, with a fitness of 30.68 doublings/h (fitness reduction of 14.41 doublings/h), no doubt primarily due to the deleterious effect of the 25-amino-acid readthrough product on gene *8*. Otherwise, fitness values remained relatively high. The double knockout strains presented the lowest fitness values. The largest reduction we observed was 17.92 doublings/h in the Δϕ9/ϕ10_8_Δstop__ strain, which had a fitness of 27.16. The additional gain in fitness reductions diminished when combining all attenuation designs. This effect is most notable when comparing the fitness of the double knockout in the wild-type background with the fitness of the double knockout in the codon-deoptimized background (Fig. 1).

10.1128/mSystems.00046-18.1 TABLE S1  Mean fitness (doublings per hour) for initial and evolved strains. Download TABLE S1, PDF file, 0.1 MB. Copyright © 2018 Paff et al. 2018 Paff et al. This content is distributed under the terms of the Creative Commons Attribution 4.0 International license.

### Promoter knockout reduces RNA expression.

Given the high expression of scaffold and capsid proteins, we expected that ablation of the ϕ9 and ϕ10 promoters would have profound effects on RNA expression not only for those genes, but also for genes *11* and *12*, as the next downstream gene with its own promoter is gene *13*. To test for changes in expression, total RNA from T7-infected E. coli cells was sequenced for all but two of the promoter knockout strains at 9 min postinfection. (Phage lysis occurs at ~11 to 12 min.) At 9 min, roughly 50 T7 proteins are expressed at detectable levels (25). Gene *10* is expressed in two forms, *10A* and *10B*. Form *10A* encodes the major capsid protein. Form *10B* (encoding the minor capsid protein) is not essential, and its expression results from a ribosomal frameshift at the end of *10A*. During RNA sequencing analysis, most fragments coming from genes *10A* and *10B* ambiguously mapped to both genes. Since the abundances of *10A* and *10B* were not differentiated using our methods, we combined them into a single *10A* measurement (and excluded sequences that mapped only to the short part of *10B* that does not overlap *10A*). Further, because we expected promoter ablation to have at most minor effects on the transcript abundance of upstream genes but to have major effects on abundances of downstream genes, we normalized transcript abundances to the total abundance of all genes up to and including *7.7*.

**FIG 2:**
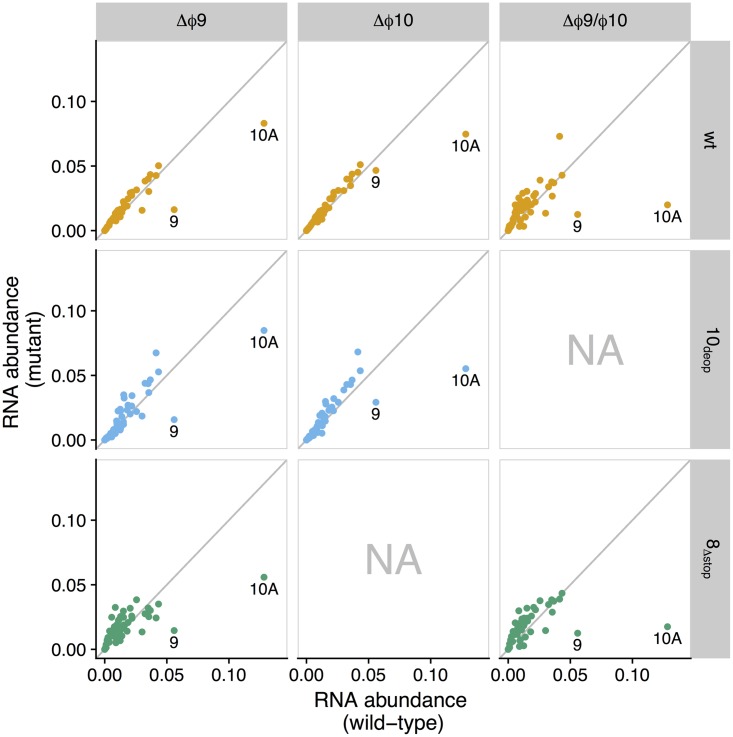
Differential gene expression for promoter knockout strains against wild type. Shown is RNA abundance (measured as transcripts per million [tpm], rescaled to a range of 0 to 1) for promoter knockout strains (*y* axis) versus wild type (x axis). Each point represents the RNA abundance for a single gene (genes *9* and *10A* are labeled). Each panel shows a different comparison of mutant versus wild type, where columns indicate promoter knockout and rows represent and are colored by genetic background (orange, wt; blue, 10_deop_; green, 8_Δstop_). Panels marked “NA” represent knockout-background combinations for which no data were collected. Samples were taken at 9 min postinfection.

**FIG 3:**
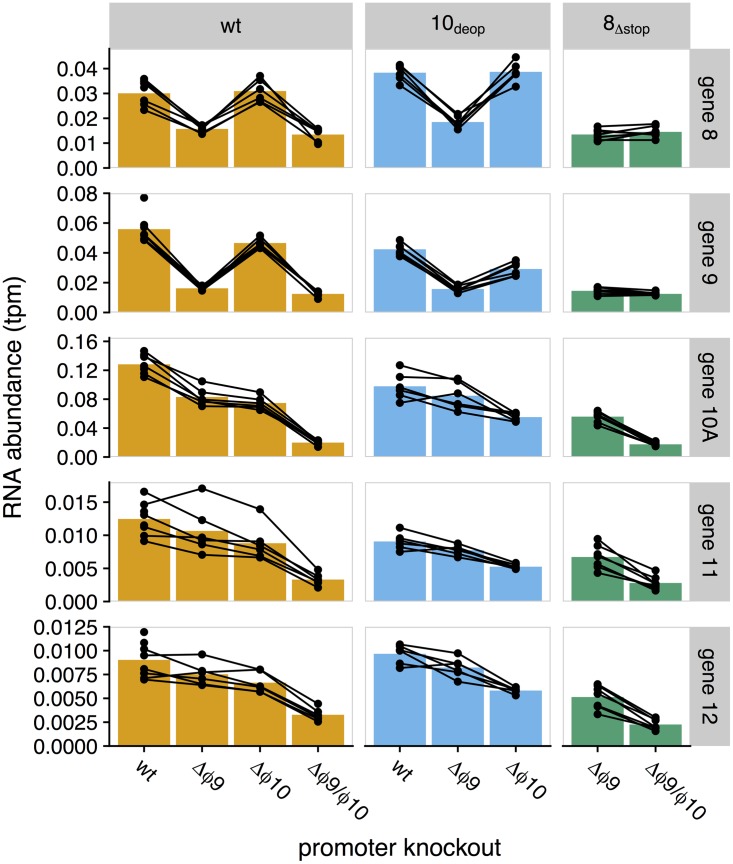
RNA abundances for genes *8* to *12*. Each bar represents the mean mRNA expression level from promoter knockout strains for genes immediately surrounding the ϕ9 and ϕ10 locations. Each point represents a single measurement. Lines connecting points indicate single batches (samples collected and sequenced together). Promoter knockouts are indicated along the *x* axis with columns organized by genetic background (column names) distinguished by color (orange, wt; blue, 10_deop_; green, 8_Δstop_). Samples were taken at 9 min postinfection.

We initially compared expression between promoter knockout strains and the wt, plotting RNA abundances for each T7 gene for each of the mutant strains against the wt (Fig. 2). At a qualitative level, genes *9* and *10A* have the greatest reduction in expression relative to the wt. In addition, expression is reduced for all genes from *9* through *12*, as well as for gene *8*, which have reduced RNA expression in strains with the Δϕ9 mutation (Fig. 3).

**FIG 4:**
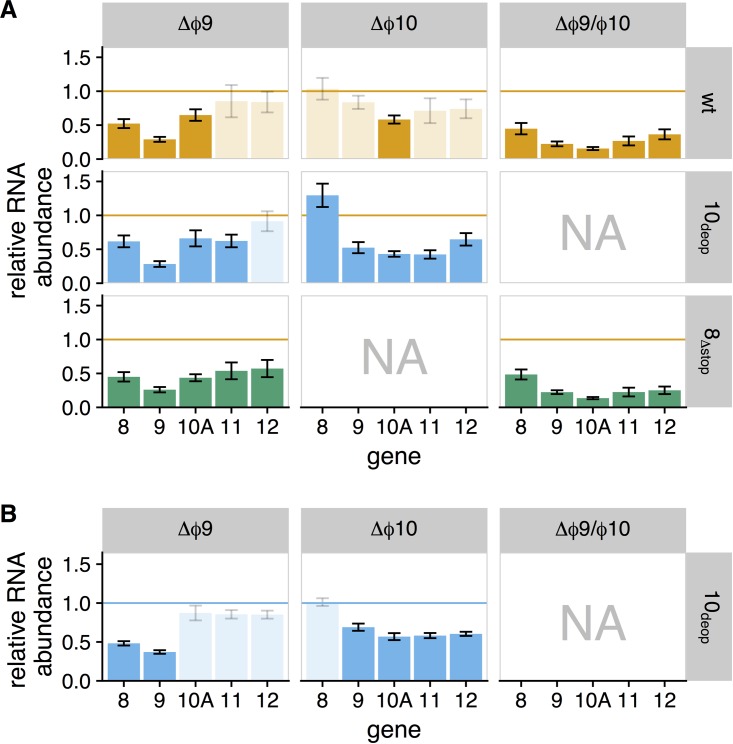
Relative RNA abundance for differentially expressed genes. Each panel represents a different strain, where columns indicate promoter knockout and rows represent genetic background. Each bar indicates the relative RNA abundance for a given gene. Genes for which a significant difference was found (FDR of <0.05) are shown as solid bars, and all other genes are shown as partially transparent bars. Panels marked as “NA” represent knockout-background combinations for which no data were collected. Colors indicate the genetic background for each strain (orange, wt; blue, 10_deop_; green, 8_Δstop_). The horizontal lines provide a reference to the ancestor strain (orange, wt; blue, 10_deop_). Bar heights below these lines indicate reduced expression, and bar heights above indicate increased expression. (A) RNA abundance relative to wild type. Adjusted *P* values for each comparison are provided in Table S2. (B) RNA abundance for 10_deop_ strains relative to wt_10_deop__. Adjusted *P* values for each comparison are provided in Table S3 in the supplemental material. Samples were taken 9 min postinfection.

Differential gene expression analysis revealed that there were indeed significant differences for genes *8* through *12* within our knockout strains. Figure 4 shows the relative RNA abundance for genes in which there was significant differential expression (false-discovery rate [FDR] of <0.05 by FDR-corrected *t* test) compared to either the wt or wt_10_deop__ (Fig. 4A and B, respectively). Generally, the differentially expressed genes had lower expression. Gene *8* RNA abundances were reduced for strains in which Δϕ9 was present. Δϕ9 had about the same effect as Δϕ10 on gene *10A* expression, and greater reductions were observed in the double knockout strains. Double knockouts had the greatest effect on gene *10A*, where transcript abundance was reduced by 84 to 86%, consistent with the expected reduction from the combined effects of the single knockouts.

10.1128/mSystems.00046-18.2 TABLE S2  Difference in relative transcript abundance between modified and wild-type strains for genes *9* to *12*. Adjusted *P* values are FDR corrected (see Materials and Methods). Download TABLE S2, PDF file, 0.1 MB. Copyright © 2018 Paff et al. 2018 Paff et al. This content is distributed under the terms of the Creative Commons Attribution 4.0 International license.

10.1128/mSystems.00046-18.3 TABLE S3  Difference in relative transcript abundance between promoter knockout and codon-deoptimized strains for genes *9* to *12*. Adjusted *P* values are FDR corrected (see Materials and Methods). Download TABLE S3, PDF file, 0.1 MB. Copyright © 2018 Paff et al. 2018 Paff et al. This content is distributed under the terms of the Creative Commons Attribution 4.0 International license.

### Promoter knockouts limit subsequent adaptation.

To evaluate the evolutionary stability of our attenuations, we carried out serial transfer adaptations on four promoter knockout strains for 30 to 35 h (~160 to 180 generations at large population size). (Evolved strains are denoted by the prefix “evo-” [see Materials and Methods].) This duration allows a beneficial mutation starting from an initial frequency of 10^−5^ to reach a frequency of 0.5 with only a fitness advantage of ~0.5 doublings/h. As T7 experimental adaptations often experience fitness increases of 10 or more doublings/h (28), lines subjected to this duration of serial transfer should have accumulated multiple large fitness mutations—if large-effect mutations are possible with these attenuations. Longer adaptations may well have realized greater fitness increases, but a short duration is presumably relevant to many uses of attenuated viruses, and the extent of fitness increase (combined with substitution identity) in the short term provides us with insight into the reversibility of the process.

**FIG 5:**
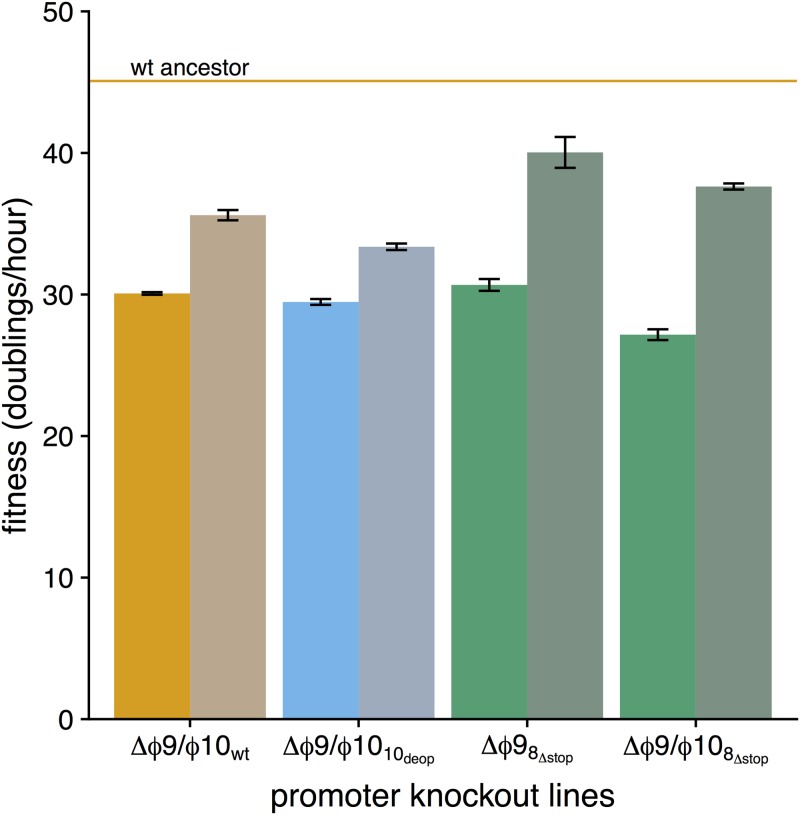
Fitness recovery for promoter knockout strains. Initial and final fitness for evolved lines after ~160 to 180 generations. Promoter knockout lines are indicated along the *x* axis, colored by genetic background (wt, orange; 10_deop_, blue; 8_Δstop_, green). Desaturated bars (on the right of each pair) indicate evolved fitness. The orange horizontal line indicates the mean fitness for the wt ancestor strain—the standard against which the magnitude of fitness recovery of attenuation is ultimately measured. Fitness increases are seen in all four lines, but all remain below wt (*P* < 0.05, two-sample *t* test). All *P* values are provided in Table S4 in the supplemental material.

Fitness improved in all four lines, although the extent of improvement varied (Fig. 5; Table S1). Increases ranged from 3.9 to 10.47 doublings/h (Δϕ9/ϕ10_10_deop__ and Δϕ9/ϕ_10_8Δstop__, respectively). Comparing recovery in relation to the wt ancestor (orange horizontal line in Fig. 5), the two recovered strains evo-Δϕ9_8_Δstop__ and evo-Δϕ9/ϕ10_8_Δstop__ had the largest relative gains in fitness, recovering 65% and 58% of initial fitness losses, respectively. The strains evo-Δϕ9/ϕ10_wt_ and evo-Δϕ9/ϕ10_10_deop__ recovered only 37% and 25% of lost fitness, respectively (Table S1).

10.1128/mSystems.00046-18.4 TABLE S4  Fitness difference between wild-type ancestor and modified strains (initial and evolved). Download TABLE S4, PDF file, 0.1 MB. Copyright © 2018 Paff et al. 2018 Paff et al. This content is distributed under the terms of the Creative Commons Attribution 4.0 International license.

Although each strain had significant increases in fitness, levels remained significantly below the ancestor strains in all cases. evo-Δϕ9_8_Δstop__ attained the highest fitness value (40.04 doublings/h) among adaptations, but was still producing 33-fold fewer phage descendants per hour than the (evolved) wild-type ancestor (Table S1). Fitness of the wild type adapted to these growth conditions is the standard for assessing whether evolution has completely recovered the fitness lost due to the attenuation.

### Sequence evolution.

In our engineered phage strains, we abolished gene-specific expression of genes *9*, *10A*, and downstream genes by replacing 18 to 23 bp corresponding to the regulatory signals for ϕ9 and/or ϕ10 with arbitrary sequences with no similarity to the canonical T7 promoter sequence. It seems that any substantial recovery of gene-specific expression would require reestablishing promoter functions for each of these genes. Such evolution is unlikely by a series of point mutations because of the many simultaneous mutations required. Promoter recovery by recombination with other promoters in the genome is a formal possibility, but there is no sequence homology to support such recombination. Any other recovery mechanisms would not be so obviously specific to expression of genes *9* and *10A*, although changes in genome-wide expression could happen through changes in the RNA polymerase gene.

To evaluate evidence of genetic evolution, genomic DNA was sequenced from initial and final populations for each of the four adaptations. Table 2 lists all mutations found to be of a frequency of ≥0.5 in each of the final evolved populations, as well as mutations fixed in the initial populations that had not previously been identified in the ancestor strains used for cloning (12, 29).

**TABLE 2:** High-frequency (≥0.5 in evolved population) nucleotide changes for all adaptations

Position	Base	Amino acid change	Gene (function)	Frequency of change in^a^:							
				Δϕ9/ϕ10_wt_		Δϕ9/ϕ10_10_deop__		Δϕ9_8_Δstop__		Δϕ9/ϕ10_8_Δstop__	
				Initial	Evolved	Initial	Evolved	Initial	Evolved	Initial	Evolved
3454	A→C	K95T	1 (RNAP)			1.00	1.00				
3835	A→C	E222A	1		0.686		0.87				
5453	T→G	I761M	1	1.00	1.00						
5483	A→G	A771A	1							1.00	1.00
9050	C→T	G51G	2 (host RNAP inhibitor)								0.735
10686	A→G	R144G	3 (endonuclease I)		0.534						
14279	T→C	I118T	4.7 (DNA metabolism)				0.061				
15124	A→G	T258A	5 (DNA polymerase)								1.00
21840	+A	Coding	8 (head-tail connector)						0.899		
21842	G→T	G535^b^	8								1.00
21927	T→C	Intergenic				1.00	1.00				
21953	G→A	A2T	9 (capsid assembly)						0.567		
22971	C→T	A2V	10A (major capsid)				1.00				
22972	T→C	A2A	10A			1.00	1.00				
22979	A→T	T5S	10A							1.00	1.00
26232	G→T	R464M	12 (tail tubular B)						0.651		
31036	T→C	S148P	16 (internal virion D)	1.00	1.00						
32350	T→C	F586L	16							1.00	1.00
35083	C→T	L154L	17 (tail fiber)					1.00	1.00	1.00	1.00
39566	C→A	Intergenic		1.00	1.00			1.00	1.00	1.00	1.00

a Evolved strains are indicated with the prefix “evo-” (e.g., evo-Δφ9/φ10_wt_) throughout the article. Because of the sequential construction of double knockouts from single knockouts, changes in the initial Δϕ9_8_Δstop__ strain would have been propagated into Δϕ9/ϕ10_8_Δstop__, as seen in the bottom two rows of the table.

b Results in nonsense mutation.

One striking observation is that new substitutions—those not present in the wild-type ancestor—appeared in the initial isolates of several engineered strains; approximately half the mutations appearing in the evolved lines were present in the ancestors. These changes could have been present in the stocks of phages used as platforms in which to introduce the knockouts, and hence irrelevant to evolutionary recovery, or they could have evolved as adaptations in response to the knockout soon after it was created. The latter explanation is plausible when realizing that what we designate as initial knockout genomes were obtained after several growth steps following disruption of the promoters—permitting adaptive evolution in response to the knockouts—and that double knockouts experienced two cycles of this opportunity for early evolution (see Materials and Methods). This second explanation is further supported in that several changes in the designated initial isolates are in genes that also experienced evolution during serial transfer of the attenuated phage (e.g., genes *1* and *10A*). The fitness of the strains designated initial isolates may thus have already increased somewhat from the step in which the promoters were first ablated (but before the *trxA* sequences were removed).

Regardless of which explanation accounts for initial substitutions, the fitness increase observed during serial transfer (in Fig. 5) is due to the substitutions that ascended then, not to changes in the initial isolate. A few outcomes are noteworthy. First, the lack of parallel evolution among lines can be interpreted as though there is no ready solution for T7 to correct the problem created by promoter knockouts. Second, all changes but one were substitutions: no deletions, duplications or insertions (larger than one base) were observed. Third, the most easily understood outcome is the one in which there was an easily reversed attenuating change with major effect: disruption of the stop codon for gene *8*. Both evolved lines carrying Δϕ9_8_Δstop__ (one line also with Δϕ10) evolved changes near the end of gene *8*. The engineering of Δϕ9 in these lines had inadvertently abolished the stop codon in gene *8*, with a consequent severe fitness defect (Fig. 1). Both mutations have the effect of introducing an earlier stop, thus correcting a major problem, and may well account for the larger recoveries seen in the Δϕ9 lines than in other lines (Fig. 5).

A common outcome was evolution in gene *1*, encoding RNAP. As noted above, several of those changes (3 of 5) appeared in the “initial” isolate of the attenuated genome, but those nonetheless may have evolved as early adaptive responses soon after the promoters were disrupted. RNAP changes at four sites were observed, scattered throughout the molecule. Changes in RNAP could have effects on transcription that differentially affect promoters, since not all promoters have an identical sequence (26), thus helping to restore a normal balance of proteins. However, we would expect changes with similar function to be clustered.

### RNA expression increases minimally in evolved populations.

The predicted basis of attenuation with promoter knockouts is reduced transcript abundance, which was observed: RNA expression for genes *9* and *10* A in the double knockouts was reduced to ~22% and ~14%, respectively, compared to the wild-type background. An obvious path to increased fitness following adaptation is recovery of transcript levels in the affected genes. However, there is no obvious mechanism for this adaptation to occur in T7, as (i) the promoter sequences require too many simultaneous mutations to evolve in brief adaptations, and (ii) there is no other clear mechanism for T7 to recover expression of just the affected genes. Fitness recovery could occur by other mechanisms, however, such as by tuning expression or activity levels of other genes, to correct imbalances (30).

**FIG 6:**
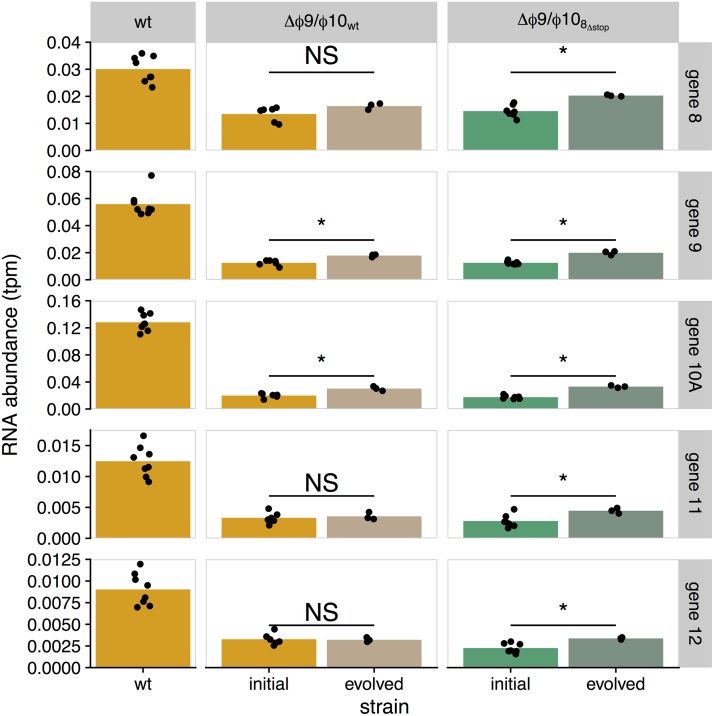
RNA abundance increases in evolved population. Shown are initial and evolved transcript abundance (measured as transcripts per million [tpm], rescaled to a range of 0 to 1) for genes *8* to *12* (rows) in 2 evolved lines (evo-Δ*ϕ*9/ϕ10_wt_, orange; evo-Δϕ9/ϕ10_8_Δstop__, green). Each point represents a single measurement, with the bars indicating the mean RNA abundances. We include transcript abundances for the wt strain (orange) as a reference. Significant increases in abundance are indicated with a star, and nonsignificant ones are labeled “NS.” Expression increases in genes *9* and *10A* in evo-Δ*ϕ*9/ϕ10_wt_ and in genes *9* to *12* in evo-Δϕ9/ϕ10_8_Δstop__ (FDR < 0.05, two-sample *t* test), but each of these genes remains well below wt levels (FDR < 0.001). Samples were taken at 9 min postinfection.

To understand mechanisms of recovery, and whether our predictions were met, levels of RNA expression were compared for initial and final populations in the evo-Δ*ϕ*9/ϕ10_wt_ and evo-Δϕ9/ϕ10_8_Δstop__ lines, again at 9 min postinfection (Fig. 6). (The wt strain is included in this figure as a reference.) Expression did increase in genes *9* and *10A* for the evo-Δ*ϕ*9/ϕ10_wt_ population and in genes 8 to 12 for evo-Δϕ9/ϕ10_8_Δstop__. However, the change in expression was minimal, and RNA abundances were still well below wt levels. Transcript abundances for genes *9* and *10A* were still at 32% and 24% of wt for evo-Δ*ϕ*9/ϕ10_wt_ and at 36% and 26% of wt for evo-Δϕ9/ϕ10_8_Δstop__ (Fig. 6). This minimal recovery of transcription is consistent with lack of new promoter evolution.

### RNA expression correlates with fitness.

**FIG 7:**
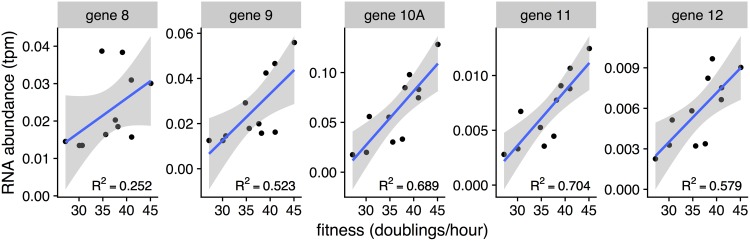
RNA abundance correlates with fitness. Shown is mean RNA abundance versus mean fitness for genes *8* to *12* with reported Pearson *R*^2^ values (0.252, 0.523, 0.689, 0.704, and 0.579, respectively). Significant positive Spearman correlations were observed for genes *10* to *12* (*P* = 0.002, 0.00006, and 0.005; FDR = 0.042, 0.003, and 0.078, respectively). Pearson correlation FDR values for the same set of genes were at least as small. A complete list of all T7 genes with both Spearman and Pearson correlations between RNA abundance and fitness can be found in the data repository (see file data/results/fitness_rna_correlation.csv in https://doi.org/10.5281/zenodo.1204715).

With parallel reductions observed in both fitness and RNA abundances between initial and evolved populations, it seemed possible that RNA expression might be used to predict fitness. We had previously reported no detectable difference in RNA expression between wild-type strains and strains attenuated by codon deoptimization (25). Our revised analysis pipeline (see Materials and Methods) revealed that codon deoptimization reduced RNA expression, but this effect was weak relative to that of the promoter knockouts. A Pearson correlation test comparing the mean RNA abundances for each gene to the mean fitnesses for each strain found significant (FDR < 0.10) correlations between RNA abundance and fitness for 34 of 59 T7 genes, including genes *8* to *12* (Fig. 7). Since the Pearson correlation test is sensitive to outliers, we then conducted a more stringent Spearman correlation test. We identified significant (FDR < 0.10) correlations for genes *10* through *12*. The Spearman *P* values for these genes were 0.85, 0.91, and 0.80, respectively (with FDR values of 0.042, 0.003, and 0.078), indicating substantial explanatory power of RNA abundance for phage fitness in the promoter knockout strains.

## DISCUSSION

This study provides insight into predicting viral attenuation using specific genome modifications (promoter deletions) and predicting evolutionary recovery of the attenuated genomes. Use of promoter knockouts has a well-understood mechanism of attenuation: reduced transcript levels and consequent reduced protein levels of the affected genes. As T7 expression generates an abundance of polycistronic, overlapping transcripts, only partial transcript suppression (rather than total abolition) is expected over the affected genes even though transcription should be totally abolished from the ablated promoters. The expected fitness effect stems from the imbalance in protein expression, which in turn should disrupt the stoichiometry of viral assembly. Re-evolution of the missing promoters by a series of point mutations should be near impossible in the short term, due to the difficulty of realizing any transcription until many of the promoter positions have attained the correct bases. Indeed, a study in which T7 was forced to grow on the RNAP of close relative T3 revealed a somewhat gradual re-evolution of promoter activity even though only 1 to 2 base changes in each T7 promoter were required to convert a T7 promoter to full recognition by T3 RNAP (29). Ectopic recombination of other T7 promoters into the knockout sites is an alternative mechanism of generating “new” promoters, but recombination in T7 is exceedingly rare unless there is sequence homology/identity between the respective flanking regions.

The results suggest we have advanced to the point of predicting qualitative attenuation and even recovery for several types of genome modifications, as long as the function of those genomic elements is well understood. Three contexts for the work are of interest.

### Attenuation by promoter knockout in the wild-type background.

The most basic alteration attempted here was the ablation of promoters for the two most highly expressed T7 genes, coding for scaffold and capsid; instead of deleting promoters, we merely replaced them with nonpromoter sequences to maintain wild-type genome spacing. Transcript levels in the altered genomes matched expectations at qualitative levels (i.e., there was no basis for quantitative predictions), with the suppression due to combined knockouts being approximately additive of the single effects. Because of the overlapping transcripts, knocking out promoters for both scaffold and capsid genes will cause an even greater suppression of capsid transcripts (the gene downstream of both promoters) than either single knockout. Despite the straightforward expectations of knockouts on transcript levels, some anomalies were observed in transcription, especially the consistent suppression of transcript levels for the gene immediately upstream of scaffold (gene *8* [head-tail connector]) whenever the scaffold promoter was ablated.

At the level of fitness, each single promoter knockout suppressed fitness moderately, and the fitness of the double knockout again was lower than expected from the combined effects of the single knockouts. Fitness of the double knockout was still high, however (almost 30 doublings/h), leaving a considerable dynamic range for further suppression.

In the wild-type background, evolutionary recovery was attempted for just the double knockout. On a log scale, approximately 30% of the initial suppression was recovered after ~150 generations of adaptation. Accompanying this fitness gain, only slight increases in transcript levels were observed for scaffold and capsid genes, and evolved changes in the genome were few. As expected, there were no evolved changes restoring the ablated promoters (i.e., too many changes would be required to restore transcription), but the evolved changes occurred in the RNA polymerase gene, highly suggestive of a global response in transcription.

The paucity of sequence changes is sometimes at odds with the fitness gains in some evolved lines. For example, the evo-Δϕ9/ϕ10_wt_ line accumulated only two new changes, neither of them nearing fixation. (The mapping detected two other changes near a frequency of 0.3, not listed because Table 2 is limited to changes above 0.5.) Regardless of how the gain in fitness from initial to evolved is partitioned among those mutations, there was more than enough time for at least one to reach near fixation. The lack of fixation suggests that there may be strong interactions among the mutations.

Overall, the molecular consequences and fitness effects of single and double promoter knockouts in the wild-type background obeyed most of the *a priori* expectations at a qualitative level. The most striking anomaly was the effect on transcript levels of a gene upstream of all promoter ablations.

### Combining attenuation designs.

Promoter knockouts were combined with a previous attenuation, codon deoptimization of the capsid gene (12). Over half the codons in the (major) capsid gene were replaced with codons encoding the same amino acid. The replacement substituted codons used at low levels in the host for codons used at high levels and hence was presumed to slow translation. The fitness impact of this deoptimization as well as the evolutionary recovery was evaluated in previous studies (12, 25), and here we merely combined this codon deoptimization with promoter knockouts.

The expected effect of combining codon deoptimization and promoter knockout is not obvious. Both designs affect the capsid gene but in different ways. The promoter knockouts reduce transcripts of the capsid gene. For codon deoptimization of the capsid gene, one of the postulated mechanisms is slowed translation, resulting in high densities of capsid transcripts stalled on ribosomes, in turn slowing down translation of all T7 genes (25). The two mechanisms may work against each other: by suppressing capsid transcript abundance, the effect of capsid codon deoptimization may be lessened.

Previous work had found little to no effect of codon deoptimization in the capsid gene on capsid transcript abundance (25). Employing a more sensitive analysis pipeline with the original data (see Materials and Methods), we found here a modest reduction in transcript abundances after deoptimization, though the effect size is smaller than that of the promoter knockouts. Thus, we expected that codon deoptimization would have little effect on transcript abundance in the genomes with promoter knockout; this was largely observed. (There was perhaps a slight reduction.) Fitness declined when we introduced codon deoptimization with a single promoter knockout but was virtually unaffected when introducing codon deoptimization with the double promoter knockout. These results add support to the model that codon deoptimization overwhelms ribosomes in the wild-type background, but only when transcript levels are high.

For the codon-deoptimized genome, evolutionary recovery was again attempted only for the promoter double knockout. As with the wild-type double knockout recovery, few changes were observed, one of them the same base change in the RNA polymerase gene (E222A) as in the wild-type T7 background. In addition, a coding change in the capsid gene was observed, this being the gene whose codons were deoptimized.

Although the combination of promoter knockout and codon deoptimization had mixed effects on fitness, one of diminishing returns, the outcomes are plausible in terms of what is understood about how the mechanisms may interact. Across all of the strains in this study, we find that transcript abundances of class III genes correlate with fitness, suggesting that even small perturbations of transcript abundances may reduce fitness. At the level of transcripts, the results are as expected.

### Unintended consequences of attenuation designs.

The rational design of attenuated genomes is usually based on simple principles: deletion of an important gene or disruption of regulation, transcription, or translation. Yet the implementation of a design from first principles invariably risks disrupting more than is intended from those principles. For example, deletion of a gene will remove the protein from the proteome but may also remove important regulatory elements, reduce genome size in the capsid, and affect structures of RNA molecules and the genome. The introduction of silent codons into a protein coding gene, intended to change codon frequencies and thus translation speed, may also change dinucleotide frequencies, RNase cleavage sites, secondary structure, and ribosome binding sites (31). To the extent these possible side effects and their relevance are understood, a genome design can achieve the intended goals while minimizing side effects. However, any design risks unintended consequences from effects we do not understand or have not anticipated, and those consequences thwart both predictions of attenuation and predictions of recovery.

Our study was subject to one unintended consequence, whose effect was fully appreciated in hindsight. When first engineering the promoter knockout for the scaffold gene (gene *9*), it was not appreciated that the stop codon for the preceding gene (gene *8* [baseplate]) was located within the ϕ9 promoter. The promoter knockout destroyed the stop, resulting in a 25-amino-acid extension of the protein. In the knockout of the scaffold promoter (wild-type background), the fitness effect of abolishing the gene *8* stop was almost three times as large as the effect of the promoter knockout *per se* (an additional decrement of 10 doublings/h on top of 4 doublings/h). The largest fitness recoveries were observed in genomes encoded with this unintended effect, and both recoveries evolved single changes that either restored the stop or created a new stop nearby.

Our specific unintended consequence serves as a model of other unintended consequences, with the advantage that we could understand it in hindsight. Attenuation that included this unintended consequence resulted in a large initial effect and rapid recovery. Avoiding this unintended consequence resulted in a more predictable and stable attenuation.

### Mechanistic models of prediction.

The broad motivation for this study is to develop models of viral life cycles that can predict the consequences of genetically altering those life cycles. Our specific focus was to rationally impair or attenuate viral fitness by a single mechanism, promoter ablation. Whereas it is trivial to impair viral fitness with virtually any genomic alteration, doing so predictively, with a clear mechanistic basis, and in a way that limits fitness recovery on extended adaptation is more challenging. Achieving such predictable attenuation likely requires a deep understanding of genetic and biochemical mechanisms underlying viral infection, replication, and assembly. T7 is ideal for such attempts because its life cycle is obligately lytic (no latency), and most of its genes are expressed by a phage-encoded RNA polymerase (26). Phages have the advantages over other viruses of easy manipulation and the simplicity of single-cell hosts.

Additionally, our study points to possible benefits of combining different mechanisms of attenuation. Use of different mechanisms may enable construction of a virus that combines a few large-effect fitness modifications with small-effect modifications that finely tune fitness. In our case, promoter knockouts may have coarse effects, whereas codon deoptimization has fine-scale effects (e.g., see references 12 and 15). Combinations may also provide different levels of protection against evolutionary reversion.

Our study follows a few important precedents in modeling the phage life cycles in light of molecular biology of the infection cycle (32–36). That work and systems approaches combining proteomics and transcription studies of phages (25) now point the way toward a new level of understanding how viral genome elements direct outcomes within the cell. At least for simple types of genome engineering, predictable attenuation—and understanding its basis and the ability of the virus to evolve escape—appears to be within reach.

Bacteriophage life cycles are certainly easier to explore than those of eukaryotic viruses—there are no cytoplasm-nuclear domains, RNA capping does not occur, and host defenses are very different—so the question might be raised as to whether results from phages can generalize to eukaryotic viruses. Our approach in this and other studies has been to investigate properties of genome organization and their relevance to fitness that we anticipate may transcend the higher taxonomic boundaries. Thus, codon modification provides fine-scale attenuation in both types of viruses, and it also retards evolutionary reversion (reviewed in reference 23). Likewise, genome rearrangement was first discovered as an attenuating mechanism in a eukaryotic virus but also functions in T7 (37–39). Partial genomic deletions are attenuating in both eukaryotic viruses and phages, and extensive recoveries have been observed in both. It is worth entertaining the possibility that mechanisms of attenuation that operate across both higher taxa of viruses may even be more evolutionarily stable than those specific to one.

## MATERIALS AND METHODS

### Strains and media. (i) Media.

Bacteria and phages were cultured in LB broth (10 g NaCl, 10 g Bacto tryptone, 5 g Bacto yeast extract/liter). Plates contained LB with Bacto agar (15 g/liter). In phage titer determination, soft agar (7 g/liter Bacto agar) was used as an overlay on LB plates.

### (ii) Bacteria.

Bacterial strains were obtained from Ian J. Molineux, and strain numbers are those from his collection. IJ1133 [E. coli K-12 F^−^ Δ*lacX74 thi*Δ (*mcrC-mrr*)*102*::Tn*10*] was used for all assays and long-term evolution experiments. HMS157 (E. coli F^−^ *recB21 recC22 sbcA5 endA gal thi sup*) (40) was used for transfections. IJ1517 (E. coli K-12 *trxA*::Kn) was used for selection of recombinant T7 strains.

### (iii) Bacteriophage.

An isolate of a previously adapted wild-type phage T7 was used for all experiments in this study, the same as in reference 12. This phage was selected for rapid growth under the serial transfer conditions also used here, so that any adaptation of the attenuated phages could be interpreted as a response to the engineering. For convenience, we will refer to this genome as wild type, although it carries several beneficial mutations that allow it to grow faster than a formal T7 wild type. Promoters for T7 gene *9* (ϕ9) and gene *10* (ϕ10) were targeted for knockout. Promoter knockout resulted in promoter sequences being replaced with a scrambled arbitrary sequence to maintain genome length (under the protocol described below). Strains were engineered to knock out either or both of the promoters within one of three genetic backgrounds (Table 1).

### Promoter knockout cloning.

The promoters for genes *9* and *10* (ϕ9 and ϕ10) in phage T7 were targeted for knockout, either individually (Δϕ9, Δϕ10) or together as a double-knockout mutant (Δ*ϕ*9/ϕ10). Promoter knockout strains were generated by replacing the wild-type promoter sequences with arbitrary sequences (Table 3) of the same length to maintain genome length. Knockouts were engineered into three different genetic backgrounds (Table 1): wt, 10_deop_, and 8_Δstop_. We refer to individual T7 strains with a notation indicating which Δ*ϕ* sequence is present and label the genetic background in the subscript (Δ*ϕ*_background_). For strains used in serial transfer experiments, the evolved population is indicated with the prefix “evo-” (evo-Δ*ϕ*_background_).

**TABLE 3:** Promoter replacement sequences

Sequence	Coordinates	Use
GAATTCCGAAGAGATTACAATAA	21848–21870	Replace ϕ9 and gene *8* stop codon
AAGCTTCGAAGAGATTACAATAA	22887–22909	Replace ϕ10
GAATTCAGAGATTACAATAA	21851–21870	Replace ϕ9 only

Promoter knockout strains were generated using a previously described protocol (29). Briefly, promoter knockout constructs were designed to contain the E. coli *trxA* gene flanked by restriction sites, followed by a nonsense sequence (to maintain genome length). This sequence was flanked on either side with 75 bp of homologous T7 sequence corresponding with the 75 bp upstream and downstream of the targeted promoter. Constructs were obtained from GenScript or IDT for synthesis and cloning into a plasmid backbone (pΔ*ϕ*). Wild-type T7 (or the phage carrying the codon-deoptimized gene *10*) was plated on IJ1133-pΔ*ϕ*, and an isolate was subsequently resuspended and replated on IJ1517 (*trxA* mutant) for selection of recombinant T7 (verified with PCR and gel electrophoresis). Following growth of an isolate to generate a phage lysate, recombinant viral DNA was then isolated, cut with appropriate restriction enzymes (EcoRI for Δϕ9 constructs and HindIII for Δϕ10 constructs), and ligated using T4 DNA ligase. Ligated DNA was transfected into HMS157 (T7-competent strain), and plaques were isolated. *trxA*-free promoter knockout isolates were confirmed via Sanger sequencing. Restriction sites unique for each promoter allowed for cloning of double knockout mutants. Double knockouts were created from two sequential knockout steps. The promoter knockout sequences used to replace the wild-type promoters are provided in Table 3.

### Serial transfers.

Promoter knockout strains were passaged as previously described (41). Frozen IJ1133 cell stocks were made by concentrating exponentially growing cells (grown in LB at 37°C), resuspending them in 20% LB–glycerol, and flash-freezing. Aliquots were stored at −80°C. Cells were thawed prior to use, added to 10 ml LB in 125-ml flasks, and grown with aeration (180 rpm) for 60 min to a density of 1 × 10^8^ to 2 × 10^8^ cells/ml before phage were added. Passaging consisted of adding 10^5^ to 10^7^ phage to the growing culture of cells and incubating them for 20 to 30 min. To maintain approximate exponential growth, between 10^5^ to 10^7^ free phage were transferred to a fresh flask of a 1-h culture of cells every 20 to 30 min. Samples from each passage were treated with chloroform to kill the remaining bacteria and release any phage particles within the cells, and the free phage was stored for future use.

Four promoter knockout lines were evolved using this protocol (Table 1). Knockout lines were passaged for 30 h (~160 to 180 generations). DNA from the initial and final populations was sequenced for molecular evolution analysis. The evo-Δϕ9/ϕ10_wt_ population was subjected to an intermediate step in which 8 sequence-verified isolates at 15 h were used to reconstitute the population, which was then subjected to an additional 20 h of adaptation. This bottleneck was introduced because the original attempted evo-Δ*ϕ*9/ϕ10_wt_ strain had experienced contamination by the Δϕ10_10_deop__ strain, and the reconstitution of sequence-verified isolates ensured exclusion of the contaminant.

### Fitness assays.

An absolute measure of fitness was used here—growth rate in the absence of a competitor. Use of a competitor may be called for when fitness effects are small, but fitness effects in this study are so large that the absolute measure is appropriate. The absolute measure also avoids complications due to interactions with the competitor and can be applied without limiting the evolved viruses to narrow dynamic ranges of fitness close to competitor fitness. Growth rate was measured by serial transfer of phage at low phage densities (where the multiplicity of infection [MOI] does not exceed 0.15) under the same conditions used for serial transfer (described above). Phage were transfered every 20 min for 5 transfers, and fitness was calculated from titers measured over the final hour (3 passages). Fitness, quantified as doublings per hour, is calculated as [log_2_(*N_t_*/*N*_0_)]/*t*, where *N*_*t*_ is the total number of phage at time *t*, adjusted for the dilution factor coming from multiple transfers (42).

### DNA sequencing.

DNA was isolated directly from phage lysates, with no DNA amplification. Purified DNA was submitted directly for library prep and sequencing (Illumina MiSeq PE 2 × 150). DNA sequencing services were provided by the University of Texas Genome Sequencing and Analysis Facility (UT GSAF) and the University of Idaho IBEST Sequencing Core. Breseq (43) was used to characterize mutations and their frequencies. Reference genome templates used to perform sequence alignments (used also for RNA sequencing alignments) were modified from the wild-type T7 genome (GenBank accession no. V01146 [26]).

### RNA sequencing.

For isolation of RNA from phage-infected E. coli samples, T7 was added at an MOI of between 2.5 and 5.0 to a 10-ml culture of exponentially growing cells. Nine minutes postinfection, 2-ml samples of phage-infected culture were collected and pelleted in a microcentrifuge. This time point was chosen based on proteomics studies of this phage (25); the T7 life cycle is short, and 9 min is as late as can feasibly be sampled without inducing lysis. Pellets were immediately resuspended in 1 ml Trizol reagent, and RNA was isolated following the manufacturer’s standard protocol. Total RNA was submitted to the UT GSAF for library prep (no RNA enrichment was performed) and sequencing (Illumina NextSeq 500 SR75). Raw fastq RNA-sequencing-reads files were aligned to the T7 genome using Hisat2 (44) to generate the corresponding *.sam files, which were converted into read count tables using Samtools (45) and Bedtools (46).

T7 is obligately lytic, and phage RNA does not reach a steady state, so we were forced to make some assumptions in our normalization of the RNA counts. Normalization to host mRNAs is not reliable, because they are being rapidly degraded during infection. Normalization to total T7 RNA results in an apparent increase in mRNA abundance of early, lowly expressed genes when later, highly expressed genes are suppressed due to promoter deletion. As the promoter deletions are located in the middle region of the T7 genome, after gene *8*, we assumed that expression of upstream genes (genes *0.3* to *7.7*) would remain unaffected. We thus first normalized the T7 RNA read counts to the sum of raw counts for genes *0.3* to *7.7* and then calculated transcripts per million (tpm), which normalizes RNA counts for gene length and read depth. This approach minimized the effect of spurious increase in mRNA abundance of early genes in proportion of the suppression of later genes due to promoter deletion, though it did not entirely eliminate it. (See negative correlations between early, lowly expressed genes and fitness in file data/results/fitness_rna_correlation.csv in the data repository at https://doi.org/10.5281/zenodo.1204715.)

To analyze differential gene expression, we performed pairwise *t* tests comparing the normalized tpm values for each gene in T7 using the Benjamini-Hochberg correction (47) with a false-discovery rate (FDR) cutoff of <0.05.

### Statistical software and visualization.

Statistical analysis was performed using the R language (48) with packages from the Tidyverse library (49). All plots were generated using the ggplot2 (50) and cowplot (51) packages.

### Data availability.

The sequencing data discussed in this article have been deposited in NCBI’s Gene Expression Omnibus (52) and are accessible through GEO series accession no. GSE115396. All processed data and analysis scripts are archived on Zenodo at https://doi.org/10.5281/zenodo.1204715. The most recent data and scripts are also available at https://github.com/mlpaff/t7-attenuation.
