# Supplementary material for: Combinatorial Approaches to Viral Attenuation
Source: mSystems. 2018 Jul 31;3(4):e00046-18. doi: 10.1128/mSystems.00046-18 (PMC6068830; doi:10.1128/mSystems.00046-18)
Supplement: TABLE S1 [file sys004182250st1.pdf]

Table S1: Mean fitness (doublings/hour) for initial and evolved strains

Strain[1]	Initial Fitness	Evolved Fitness
wt	45.08 ± 1.30	–
$\text{wt}_{10_{\text{deop}}}$	39.12 ± 0.55	–
$\Delta\phi 9_{\text{wt}}$	41.05 ± 0.12	–
$\Delta\phi 9_{10_{\text{deop}}}$	38.18 ± 0.42	–
$\Delta\phi 9_{8_{\Delta\text{stop}}}$	30.68 ± 0.42	40.04 ± 1.09
$\Delta\phi 10_{\text{wt}}$	40.99 ± 0.45	–
$\Delta\phi 10_{10_{\text{deop}}}$	34.80 ± 0.55	–
$\Delta\phi 9/\phi 10_{\text{wt}}$	30.07 ± 0.09	35.60 ± 0.36
$\Delta\phi 9/\phi 10_{10_{\text{deop}}}$	29.47 ± 0.21	33.37 ± 0.23
$\Delta\phi 9/\phi 10_{8_{\Delta\text{stop}}}$	27.16 ± 0.38	37.63 ± 0.22

[1]Each strain is labeled according to the $\Delta\phi$ sequence that is present ("wt" indicates lack of promoter knockout) with the genetic background indicated in the superscript (wt, 10_{deop}, or $8_{\Delta\text{stop}}$).
