# Supplementary material for: Combinatorial Approaches to Viral Attenuation
Source: mSystems. 2018 Jul 31;3(4):e00046-18. doi: 10.1128/mSystems.00046-18 (PMC6068830; doi:10.1128/mSystems.00046-18)
Supplement: TABLE S2 [file sys004182250st2.pdf]

Table S2: Difference in relative transcript abundance between modified and wildtype strains for genes *9–12*. Adjusted p-values are FDR corrected (see Methods).

Strain	Gene	Difference	Adjusted p-value
$\Delta\phi 9_{\mathrm{wt}}$	*9*	0.0400	2.5e-04
	8	0.0140	4.8e-04
	10A	0.0450	4.8e-04
	12	0.0015	2.6e-01
	11	0.0018	4.5e-01
$\Delta\phi 9/\phi 10_{\mathrm{wt}}$	*10A*	0.1100	3.0e-07
	9	0.0430	2.2e-05
	8	0.0170	3.2e-05
	11	0.0092	3.2e-05
	12	0.0058	9.3e-05
$\Delta\phi 10_{\mathrm{wt}}$	*10A*	0.0540	2.4e-05
	12	0.0024	9.7e-02
	9	0.0093	1.3e-01
	11	0.0037	1.3e-01
	8	−0.0009	8.0e-01
$\Delta\phi 9_{\mathrm{deop}}$	*9*	0.0400	4.5e-05
	8	0.0120	1.7e-03
	11	0.0047	4.3e-03
	10A	0.0430	5.5e-03
	12	0.0008	4.6e-01
$\Delta\phi 10_{\mathrm{deop}}$	*10A*	0.0730	1.4e-06
	9	0.0270	4.6e-04
	11	0.0072	9.4e-04
	12	0.0032	7.4e-03
	8	−0.0086	1.2e-02
$\Delta\phi 9_{8_{\Delta\mathrm{stop}}}$	*10A*	0.0720	6.0e-07
	9	0.0410	3.0e-05
	8	0.0170	6.0e-05
	11	0.0057	8.7e-04
	12	0.0039	1.2e-03
$\Delta\phi 9/\phi 10_{8_{\Delta\mathrm{stop}}}$	*10A*	0.1100	6.0e-07
	9	0.0430	3.0e-05
	11	0.0097	3.0e-05
	12	0.0068	6.8e-05
	8	0.0160	7.5e-05
