# Supplementary material for: Combinatorial Approaches to Viral Attenuation
Source: mSystems. 2018 Jul 31;3(4):e00046-18. doi: 10.1128/mSystems.00046-18 (PMC6068830; doi:10.1128/mSystems.00046-18)
Supplement: TABLE S3 [file sys004182250st3.pdf]

Table S3: Difference in relative transcript abundance between promoter knockout and codon-deoptimized strains for genes *9–12*. Adjusted p-values are FDR corrected (see Methods).

Strain	Gene	Difference	Adjusted p-value
$\Delta\phi9_{\text{deop}}$	*8*	0.02000	2.5e-05
$\Delta\phi9_{\text{deop}}$	*9*	0.02700	2.5e-05
$\Delta\phi9_{\text{deop}}$	*12*	0.00140	2.1e-01
$\Delta\phi9_{\text{deop}}$	*11*	0.00130	2.8e-01
$\Delta\phi9_{\text{deop}}$	*10A*	0.01300	6.1e-01
$\Delta\phi10_{\text{deop}}$	*9*	0.01300	6.2e-03
$\Delta\phi10_{\text{deop}}$	*11*	0.00380	6.2e-03
$\Delta\phi10_{\text{deop}}$	*12*	0.00390	6.2e-03
$\Delta\phi10_{\text{deop}}$	*10A*	0.04300	1.4e-02
$\Delta\phi10_{\text{deop}}$	*8*	-0.00033	9.2e-01
