# Supplementary material for: Combinatorial Approaches to Viral Attenuation
Source: mSystems. 2018 Jul 31;3(4):e00046-18. doi: 10.1128/mSystems.00046-18 (PMC6068830; doi:10.1128/mSystems.00046-18)
Supplement: TABLE S4 [file sys004182250st4.pdf]

Table S4: Fitness difference between wildtype ancestor and modified strains (initial and evolved).

Strain	Estimate	p-value
$\Delta\phi 9/\phi 10_{\mathrm{wt}}$ (initial)	-15.010000	0.0071805
$\Delta\phi 9/\phi 10_{\mathrm{wt}}$ (evolved)	-9.480000	0.0132091
$\Delta\phi 9/\phi 10_{10_{\mathrm{deop}}}$ (initial)	-15.610000	0.0058612
$\Delta\phi 9/\phi 10_{10_{\mathrm{deop}}}$ (evolved)	-11.713333	0.0102897
$\Delta\phi 9_{8_{\Delta\mathrm{stop}}}$ (initial)	-14.406667	0.0043853
$\Delta\phi 9_{8_{\Delta\mathrm{stop}}}$ (evolved)	-5.045833	0.0366923
$\Delta\phi 9/\phi 10_{8_{\Delta\mathrm{stop}}}$ (initial)	-17.923333	0.0029610
$\Delta\phi 9/\phi 10_{8_{\Delta\mathrm{stop}}}$ (evolved)	-7.455833	0.0263225
